# CMR myocardial tissue characterization in a mouse model of doxorubicin-induced cardiotoxicity

**DOI:** 10.1186/1532-429X-17-S1-Q135

**Published:** 2015-02-03

**Authors:** Bradley D Allen, Micah Anderle, Sol Misener, Gillian Murtagh, Nicholas Furiasse, Nausheen Akhter, Daniele Procissi, James C Carr

**Affiliations:** 1 Radiology, Northwestern University, Chicago, IL, USA; 2 Medicine-Cardiology, Northwestern University, Chicago, IL, USA

## Background

Cardiotoxicity is a major limiting factor preventing dose-effective use of multiple cytotoxic agents in cancer treatment. Current cardiotoxicity diagnosis is based on functional changes that occur late in the course of the disease. In this pre-clinical pilot study, we hypothesize myocardial T1 and T2 mapping will identify mice at risk of death secondary to doxorubicin-induced cardiotoxicity prior to cardiac function deterioration.

## Methods

**Figure 1:**
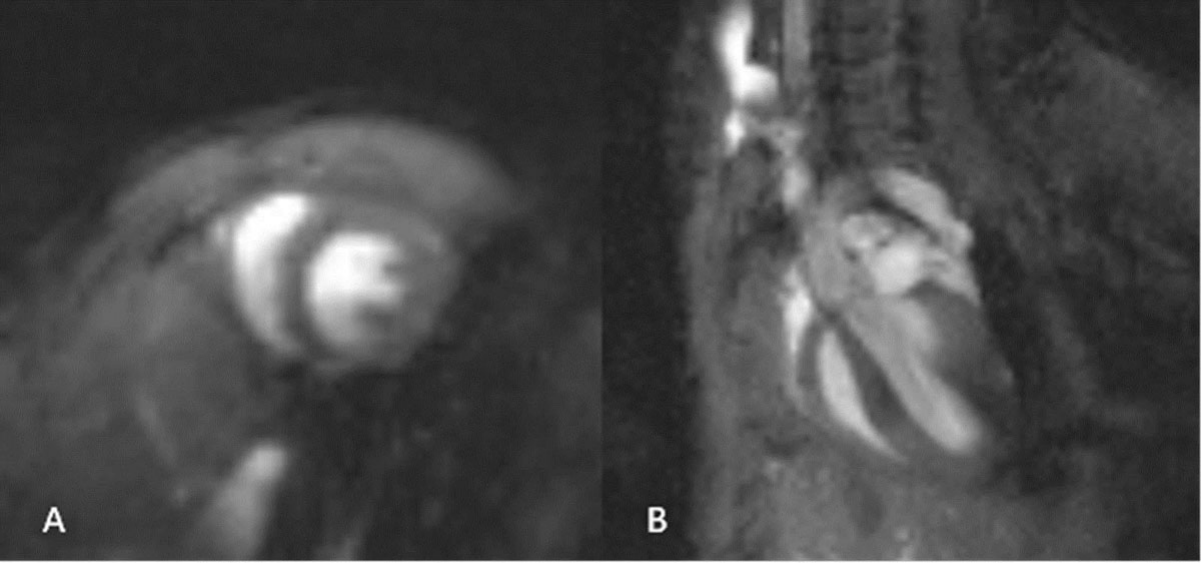
Cardiac MR at 7 Tesla in a mouse model of cardiotoxicity. Representative short axis (A) and 3-Chamber (B) balanced steady-state free precession (bSSFP) cine images. The short axis images acquired at multiple slices along the left ventricle are used to calculate ejection fraction as an assessment of cardiac function.

Eleven healthy, 10-week-old C57BL/6 female mice were included in the study. All procedures are in accordance with an Institutional Animal Care and Use Committee approved protocol. Doxorubicin-induced cardiotoxicity was generated by giving 8 mg/kg of doxorubicin via intraperitoneal (IP) injection weekly for 4 weeks in n = 8 randomly selected mice. A control group of n = 3 mice was given a weekly volume equivalent dose of IP normal saline. Each mouse underwent ECG-gated CMR on a 7T small animal scanner (ClinScan, Bruker, Billerica, MA) prior to treatment, at 2 weeks, and post-treatment. Steady-state free precession images were acquired at the left ventricular base, mid-ventricle, and apex and ejection fraction (EF) was calculated (Figure 1). Gadopentetate Dimeglumine contrast was injected via the tail vein at a dose of 1 mL/kg. Myocardial T1 and T2 maps were generated using dedicated offline software to calculate the pixel-wise relaxation times at a mid-left ventricle short axis slice. An inversion recovery sequence with variable flip angles (α = 3^o^, 6^o^, 10^o^, 16^o^, and 25^o^) was used for T1 mapping and a spin-echo sequence with varied echo times (TE = 7 ms, 14 ms, 21 ms, 28 ms) was used for T2 mapping. Average myocardial T1 and T2 relaxation times and ejection fraction were compared between mice that died during treatment, survived to the end of treatment, and controls using repeated measure linear modeling.

## Results

**Figure 2:**
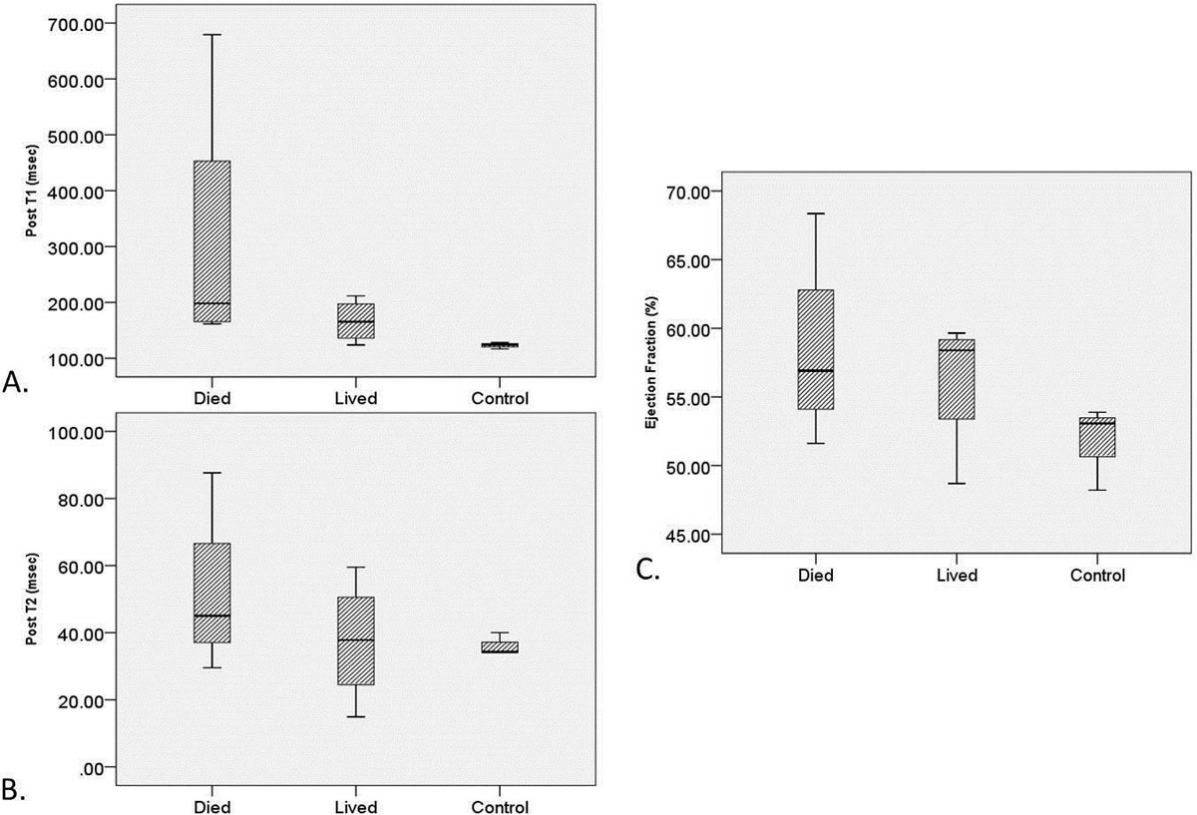
Pre-treatment average myocardial T1 (A) and T2 (B) relaxation times and ejection fraction (C) in doxorubicin-treated mice that died, lived, and control mice. Note the trend of increased, high-variability T1 and T2 values in treatment mice that died relative to those who survived the doxorubicin course and control mice.

In the treatment group, n = 4 mice survived and n = 4 mice died during treatment. All control mice survived. Linear modeling demonstrated a significant relationship between T2 relaxation times and outcome when controlling for pre-, intra-, or post-treatment scan (β = -0.008, p = 0.02). T1 (β = -0.001, p = 0.10). and EF (β = -0.015, p = 0.09) did not demonstrate a significant relationship. There was a non-significant trend of increased pre-treatment myocardial T1 (309 ± 248 ms vs. 167 ± 38 ms, p = 0.29) and T2 (52 ± 25 ms vs. 38 ± 19 ms, p = 0.49) in mice who died compared to those who survived. EF doesn't show the same trend (58 ± 7% vs. 56 ± 5%). (Figure 2)

## Conclusions

Average myocardial T2 in mice undergoing IP chemotherapy was significantly correlated with outcome, while T1 and EF showed no correlation. The trends identified in this pre-clinical pilot study further drive the hypothesis that myocardial tissue characterization may be an effective tool for diagnosis and risk-stratification of chemotherapy-induce cardiotoxicity.

## Funding

Northwestern CTI Pilot Grant, NIH/NCI R25 CA 132822.

